# Genomics of Hairy Cell Leukemia

**DOI:** 10.1200/JCO.2016.71.1556

**Published:** 2017-02-13

**Authors:** Enrico Tiacci, Valentina Pettirossi, Gianluca Schiavoni, Brunangelo Falini

**Affiliations:** All authors: Institute of Hematology and Center for Hemato-Oncology Research, University and Hospital of Perugia, Perugia, Italy.

## Abstract

Hairy cell leukemia (HCL) is a chronic mature B-cell neoplasm with unique clinicopathologic features and an initial exquisite sensitivity to chemotherapy with purine analogs; however, the disease relapses, often repeatedly. The enigmatic pathogenesis of HCL was recently clarified by the discovery of its underlying genetic cause, the BRAF-V600E kinase-activating mutation, which is somatically and clonally present in almost all patients through the entire disease spectrum and clinical course. By aberrantly activating the RAF-MEK-ERK signaling pathway, BRAF-V600E shapes key biologic features of HCL, including its specific expression signature, hairy morphology, and antiapoptotic behavior. Accompanying mutations of the KLF2 transcription factor or the CDKN1B/p27 cell cycle inhibitor are recurrent in 16% of patients with HCL and likely cooperate with BRAF-V600E in HCL pathogenesis. Conversely, BRAF-V600E is absent in other B-cell neoplasms, including mimickers of HCL that require different treatments (eg, HCL-variant and splenic marginal zone lymphoma). Thus, testing for BRAF-V600E allows for a genetics-based differential diagnosis between HCL and HCL-like tumors, even noninvasively in routine blood samples. BRAF-V600E also represents a new therapeutic target. Patients’ leukemic cells exposed ex vivo to BRAF inhibitors are spoiled of their HCL identity and then undergo apoptosis. In clinical trials of patients with HCL who have experienced multiple relapses after purine analogs or who are refractory to purine analogs, a short course of the oral BRAF inhibitor vemurafenib produced an almost 100% response rate, including complete remission rates of 35% to 42%, without myelotoxicity. To further improve on these results, it will be important to clarify the mechanisms of incomplete leukemic cell eradication by vemurafenib and to explore chemotherapy-free combinations of a BRAF inhibitor with other targeted agents (eg, a MEK inhibitor and/or an anti-CD20 monoclonal antibody).

## INTRODUCTION

Hairy cell leukemia (HCL) is a chronic peripheral B-cell lymphoid neoplasm recognized as a distinct nosologic entity by the WHO classification of hematologic malignancies.^1^ Although the incidence of HCL is low (approximately 0.3 cases per 100,000 persons per year, corresponding to approximately 1,400 new patients expected annually in Europe^2,3^), its prevalence is considerably higher (approximately 15,000 patients in 2008 in Europe^2,3^) because most patients respond well to chemotherapy with purine analogs (cladribine and pentostatin) but are not cured and tend to experience repeated relapses over time.^4^ HCL is four to five times more frequent in men than women (for unknown reasons) and usually presents in 50- to 60-year-old patients with pancytopenia (including monocytopenia), splenomegaly, and no lymphoadenopathy.^1^ Bone marrow, spleen, and liver are infiltrated by mature B cells that usually circulate in low numbers in the blood and show a peculiar morphology (ample cytoplasm with thin surface projections, giving the disease its name^5^) and a specific surface immunophenotype (coexpression of CD103, CD25, and CD11c^1^). Despite the unique clinicopathologic features of HCL, which were first described in 1958,^6^ its genetic cause has remained enigmatic for more than 50 years, partly because of the absence of faithful cell line or mouse models of this disease^7-9^ and partly as a result of the difficulty of recovering enough primary tumor cells for analysis from the marrow (often inaspirable as a result of HCL-induced fibrosis^1^) or the blood (often containing few leukemic cells).

## EARLIER GENOMIC STUDIES

Nevertheless, genome-wide studies analyzing the expression of protein-coding and microRNA genes^10,11^ were successfully performed and unraveled a transcriptional signature specific of HCL that provided important insights into its putative cell of origin (ie, a germinal center–experienced memory B cell) and into some of its biologic properties (eg, the typical morphology, the bone marrow fibrosis, and the selective dissemination pattern to certain anatomic sites).^12^

Furthermore, several studies attempted to clarify the genetics of HCL through a variety of targeted and genome-wide, low- and high-resolution techniques, such as cytogenetics, fluorescence in situ hybridization, array comparative genomic hybridization, and single-nucleotide polymorphism genotyping. Yet, the HCL genome turned out to be remarkably stable and balanced; no recurrent chromosomal translocations were identified, and no copy number aberrations were consistently detected at significant frequencies, with the possible exception of deletions affecting the long arm of chromosome 7 in less than 10% of total patients.^13-19^ However, all of these methodologies, although suitable for identifying structural and numerical DNA alterations, are not geared to interrogate the DNA sequence at the nucleotide level.

## THE BRAF-V600E MUTATION AS THE GENETIC CAUSE OF HCL

**Fig 1.**
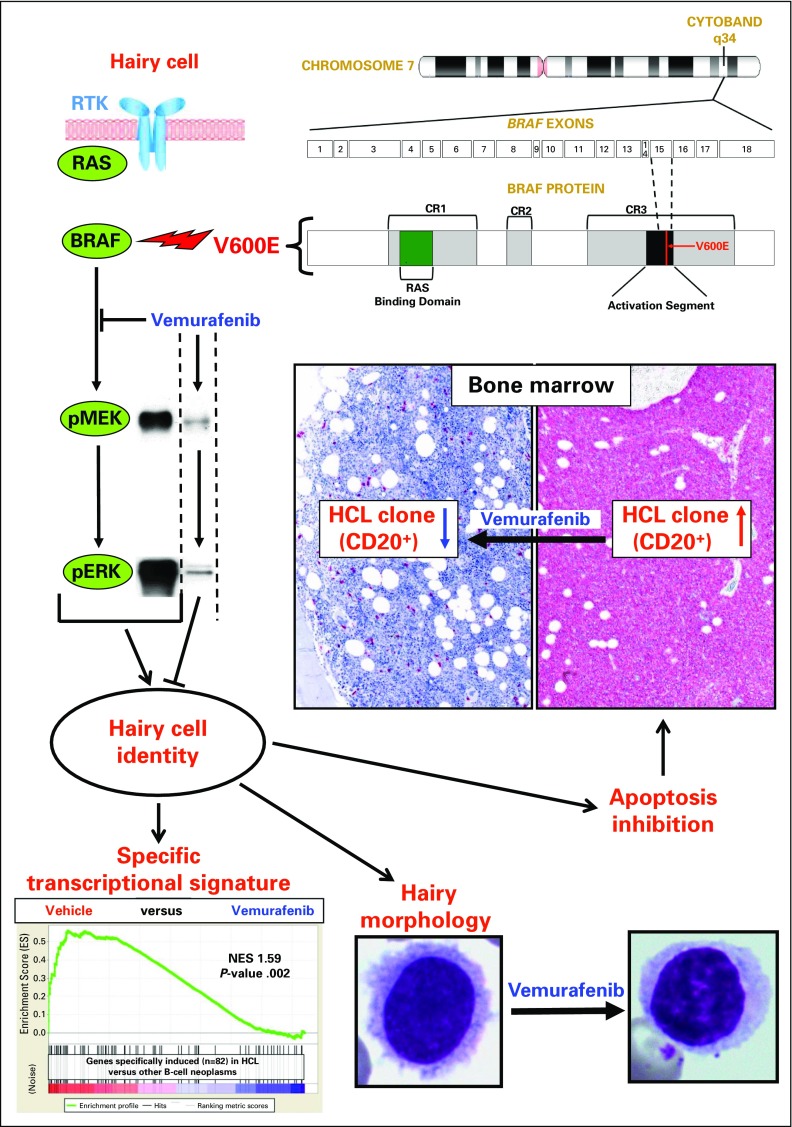
The BRAF-V600E mutation and hairy cell leukemia (HCL) pathogenesis. In the top right of the figure, an ideogram of chromosome 7 is shown indicating the cytoband q34, which contains the *BRAF* gene with its 18 exons displayed below. Further below, a scheme of the BRAF protein is displayed, with the V600E mutation (encoded by exon 15) occurring in the kinase activation segment. Other protein domains are also indicated (conserved region [CR] 1-3; CR3 is the kinase domain). In the top left and middle left part of the figure, a hairy cell is outlined harboring the V600E mutation of the BRAF kinase, which makes its enzymatic activity constitutive and uncoupled from upstream regulators (receptor tyrosine kinases [RTKs] and RAS). The consequent aberrant phosphorylation of MEK1/2 (pMEK) and ERK1/2 (pERK) downstream of BRAF-V600E (Western blot bands in the middle left) can be blocked by the BRAF inhibitor vemurafenib and drives the acquisition of the distinct HCL identity. The latter includes a specific gene expression signature, which vemurafenib silences (gene set enrichment analysis plot in the bottom left), as well as a typical hairy morphology and an antiapoptotic behavior that are both sequentially reverted by BRAF blockade. In particular, in the bottom right, blood smears from a patient with HCL show rich hair-like surface projections in a leukemic cell at baseline but not 2 days after starting oral vemurafenib intake. Apoptosis inhibition (middle right) causes the cancer clone to expand in the spleen (not shown) and the bone marrow; the massive leukemic infiltration on the right (highlighted in red by the immunostaining with the B-cell marker CD20) can be largely cleared, and a normal hematopoiesis restored, after treatment with vemurafenib (producing, on the left, a complete remission after 8 weeks of treatment, but with some persisting leukemic cells). The gene set enrichment analysis plot in the bottom left of this figure is reprinted with permission.^21^ Copyright American Society of Hematology. The blood smear micrographs in the bottom right of this figure are reprinted with permission.^22^ Copyright Massachusetts Medical Society.

The advent of massively parallel sequencing made it possible in 2011 to discover, starting from the whole-exome analysis of just one patient with HCL,^20^ that the causal genetic lesion of this cancer was a single somatic, clonal, point mutation in the DNA sequence of *BRAF*, a kinase-encoding proto-oncogene that, at the time, was little known in hematologic malignancies. The mutation consists of the replacement of a thymine (T) with an adenine (A) in exon 15 of *BRAF* at position 1799 of the gene-coding sequence located in chromosome 7q34. In turn, this produces an amino acid change from valine (V) to glutamate (E) at position 600 (V600E) of the protein sequence (Fig 1, top right), ultimately leading to aberrant activation of the BRAF oncogenic kinase and, thus, of the downstream MEK-ERK signaling pathway^23^ (Fig 1, top left and middle left). The BRAF-V600E mutation in HCL is heterozygous, except in a minority of patients who lose the wild-type allele as a result of 7q deletions.

The first report^20^ and a number of subsequent studies from different groups worldwide,^19,24-37^ which included several hundred patients, have confirmed that the recurrency of this mutation in patients with HCL approaches 100%. Exceptional non-V600E BRAF mutations have been described in only two patients so far, both in exon 11 (ie, F468C, which is known to be activating, and D449E, which is of unclear functional relevance).^38^ In one series, the BRAF-V600E mutation frequency was lower (79%, in 53 patients)^39^ than in all other series.^19,20,24-37^ However, in this study,^39^ there was a bias toward patients seeking clinical trials for relapse after or refractoriness to purine analogs. Indeed, in these *BRAF* wild-type patients, leukemic cells often carried a particular unmutated or lowly mutated immunoglobulin heavy chain variable gene rearrangement (*IGHV4-34*)^39^ that is typical of HCL-variant,^40^ a mimicker of HCL that responds poorly to purine analogs.^41^ Consistently, the unmutated or lowly mutated *IGHV4-34* rearrangement associates with clinical and genetic features similar to those of HCL-variant, including higher WBC counts at diagnosis, low response to cladribine, and frequent activating mutations of *MAP2K1*/*MEK1*^40,^^42^^,^^43^ (encoding the kinase phosphorylated by BRAF).

The BRAF-V600E mutation has all the hallmarks of the disease-defining genetic lesion in HCL.^19,20,24-37^ Indeed, it is clonally and somatically present at diagnosis in almost all patients across the entire clinical spectrum of the disease, including patients presenting with leukemic lymphocytosis or without splenomegaly, and it has been detected in anatomic sites rarely involved by HCL (eg, lymph nodes). Moreover, the BRAF-V600E mutation is extremely stable over the whole disease course, including after multiple relapses even decades after initial presentation. Finally, the BRAF-V600E mutation is quite specific for HCL among B-cell neoplasms, including mimickers of HCL such as HCL-variant and splenic marginal zone lymphoma.^20,24,27,44-46^ It is also worth noting that, whereas mutations of nonkinase genes are most prevalent as driving events in mature B-cell tumors, HCL stands out in that its key genetic lesion activates a kinase-encoding gene.^20^

## THE BRAF-V600E MUTATION AND HCL PATHOGENESIS

BRAF is a serine-threonine kinase of the RAF family (also comprising RAF1/CRAF and ARAF) and a key component of the RAS–RAF–MEK-ERK signaling pathway^23^ (Fig 1, top left and middle left). This cascade transduces within the cell survival and proliferation signals coming in a controlled way from surface receptors (including receptor tyrosine kinases) only when engaged by their cognate ligands. Ligand-stimulated receptors cause activation of membrane RAS, which recruits cytosolic RAFs to the plasma membrane. This in turn favors phosphorylation of RAFs in their activation segment and RAF dimerization. Active RAFs then phosphorylate and activate MEK1 and MEK2 kinases, which in turn phosphorylate and activate ERK1 and ERK2 kinases. ERKs disseminate the signal within the cell by phosphorylating in the cytoplasm and nucleus hundreds of targets, including various transcription factors, that elicit the pathway response. However, ERKs also phosphorylates RAFs themselves at specific inhibitory amino acid residues, which releases RAFs from RAS and extinguishes the signal via a negative feedback mechanism.^23^ In this way, ERK-dependent phosphorylation can direct in a controlled way a wide range of cellular responses, among which growth, proliferation, and survival are key in cancer pathogenesis when they become deregulated.

The BRAF-V600E mutation, which is also recurrent in various solid tumors (eg, cutaneous melanoma),^47^ occurs in the kinase activation segment (Fig 1, top right) and mimics its phosphorylation independently from upstream RAS (Fig 1, top left), resulting in constitutive kinase activity^48^ and aberrant signaling through the RAF-MEK-ERK pathway^20,49^ (Fig 1, middle left). Indeed, ex vivo and in vivo human studies have shown that HCL cells are characterized by high levels of MEK and ERK phosphorylation and that these levels are drastically reduced by treatment with small-molecule inhibitors of active BRAF (eg, vemurafenib or dabrafenib; Fig 1, middle left).^20,21,49-51^ Inhibitor-induced MEK and ERK dephosphorylation in HCL cells not only silences the transcriptional output of the BRAF-MEK-ERK pathway as defined in BRAF-V600E–positive solid tumors, but also downregulates the HCL-specific expression signature^10^ (Fig 1, bottom left), including some immunophenotypic markers (cyclin D1, tartrate-resistant acid phosphatase, and CD25)^21^ that are routinely used for the differential diagnosis of HCL from other B-cell malignancies. Furthermore, as BRAF inhibition proceeds, HCL cells (but not HCL-variant cells) lose their surface protrusions while still being alive (Fig 1, bottom right) and eventually undergo apoptosis^21^ (Fig 1, middle right); this is consistent with the fact that apoptosis inhibition is considered the main tumor growth mechanism in HCL, being that its proliferative index (< 5%) is one of the lowest among B-cell neoplasms.^52^ In other words, leukemic cells seem to rely heavily on the BRAF-V600E mutation for most of their unique molecular, morphologic, and biologic features, such that BRAF blockade dramatically spoils tumor cells of their distinctive HCL identity and viability (Fig 1). The striking extent of this phenomenon, which could not be anticipated from studies on *BRAF*-mutated solid tumors,^47^ is likely a result, at least in part, of the lower complexity of the HCL genome as compared with the much greater burden of genetic lesions typical of solid tumors.

The fine molecular mechanisms through which BRAF-V600E governs the various facets of HCL biology and the importance of the latter to leukemogenesis remain to be worked out. For example, it is unclear how the hairy morphology is mechanistically imparted by mutant *BRAF* and whether hairiness is just an irrelevant epiphenomenon of neoplastic transformation or whether it could teleologically benefit leukemic cells in some way (eg, by augmenting the surface area susceptible to microenvironmental signals that increase cellular fitness). Another fascinating and unresolved topic is the observation that, in patients with HCL, the BRAF-V600E mutation occurs as early as in hematopoietic stem and progenitor B cells, endowing them with enhanced clonogenic potential.^9^ However, the fully blown HCL phenotype apparently develops only after these mutated cells have traversed a long series of differentiation steps in the bone marrow and peripheral lymphoid organs and have eventually become memory B cells, to which HCL resembles the most both transcriptionally and histogenetically (as a result of its mutated immunoglobulin gene rearrangements in the vast majority of cases).^10,12^ Whether complete development of the HCL identity requires additional genetic events along the way and/or a permissive epigenetic landscape specific of a particular cell differentiation stage is not fully clear. On the one hand, recurrent mutations accompanying BRAF-V600E are found in a minority of patients with HCL (see discussion in next section), but on the other hand, pharmacologic blockade of BRAF-V600E in the established leukemic clone seems sufficient to erase several key specific traits of HCL.^21,22^

## OTHER GENES RECURRENTLY MUTATED IN HCL

Recently, targeted genetic analyses revealed somatic mutations or deletions, mostly clonal and monoallelic, of the *KLF2* gene in 31% of splenic marginal zone lymphomas (a mimicker of HCL^53^) and 26% of diffuse large B-cell lymphomas.^54^ *KLF2* mutations were also observed in four (16%) of 24 patients with HCL and, at lower frequencies, in a variety of other lymphoid neoplasms, including nodal and extranodal marginal zone lymphomas.^54^ KLF2 is a transcription factor controlling the homeostasis and differentiation of multiple mature B-cell subpopulations, including marginal zone B cells,^54^ a compartment where memory B cells can also be found.^12^ *KLF2* mutations in HCL led to amino acid replacements, whereas in other tumors, these mutations also included clearly destructive variants (ie, nonsense mutations, frameshift mutations, or mutations involving conserved splice sites). In this study of *KLF2* mutations^54^ and in a related study,^55^ functional experiments on a few missense and truncating *KLF2* alleles documented the loss-of-function character of all of them, except one encoding for an amino acid replacement (A291V). The clonal representation, the functional consequences, and, in one instance, the somatic status of the missense variants specifically found in HCL are unclear,^54,55^ and further studies are therefore needed to better clarify the role of *KLF2* mutations in HCL pathogenesis.

Through a subsequent whole-exome sequencing study,^19^ mutations of the tumor suppressor *CDKN1B* gene, encoding for the cyclin-dependent kinase inhibitor p27, were identified in 13 (16%) of 81 patients with HCL. Mutations were mostly clonal, disruptive, and monoallelic, suggesting haploinsufficiency for tumor suppression, and were not found to impact the clinical characteristics of patients or their outcome. The transforming potential of oncogenes, including mutant *BRAF*, can be counteracted by cell cycle arrest and senescence as protective fail-safe mechanisms, such that genetic inactivation of cell cycle inhibitors is often selected for during cancer pathogenesis.^56^ In HCL, p27 protein expression is absent or weak in 100% of patients,^52^ pointing to additional mechanisms of *CDKN1B* silencing beyond gene mutations. Thus, downregulation of p27 activity, through genetic disruption^19^ and/or reduction of protein expression^52^ potentially induced by BRAF-MEK-ERK signaling itself,^57^ may facilitate HCL clonal expansion driven by mutant *BRAF*. It is worth noting that *CDKN1A*/p21, another cyclin-dependent kinase inhibitor promoting senescence,^58^ can be a direct target of the KLF2 transcription factor,^59,60^ which is also mutated (and perhaps functionally impaired) in some patients with HCL.

## THE BRAF-V600E MUTATION AND HCL DIAGNOSIS

**Fig 2.**
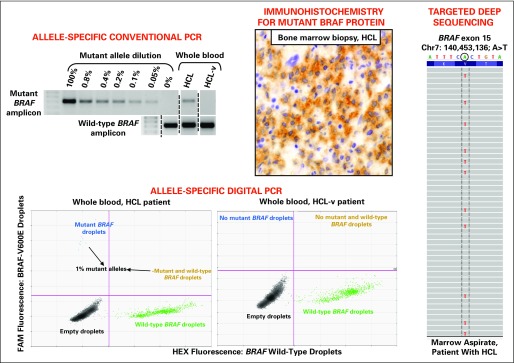
The BRAF-V600E mutation and hairy cell leukemia (HCL) diagnosis. Establishing the presence or absence of the BRAF-V600E mutation and, in this way, distinguishing HCL from its mimickers can be accomplished in various types of biologic specimens by multiple techniques. For example, conventional (top left panel) and digital (bottom panels) allele-specific DNA polymerase chain reaction (PCR) are sensitive enough to detect even rare mutant alleles (approximately 1% here) in unfractionated blood samples of patients with HCL but not in those with HCL-variant (HCL-v), which enables a genetic diagnosis of HCL noninvasively in the blood (where HCL cells are usually scarce); presence of the *BRAF* wild-type amplicon acts as positive control in samples lacking the mutation. In the middle top panel, the BRAF-V600E amino acid replacement can be detected by a monoclonal antibody specific for the mutant epitope (clone VE1 in the example shown), which stains in brown the ample cytoplasm of leukemic cells in a fixed bone marrow biopsy from a patient with HCL. In the right panel, a pileup of reads (in gray) generated by targeted deep sequencing of *BRAF* exon 15 in a marrow aspirate from a patient with HCL shows the pathognomonic point mutation (red T *v* green A of the reference human genome above) in a proportion of reads, reflecting the fraction of mutant alleles present in the sample; note that the mutation appears as A to T (instead of T to A) because *BRAF* is conventionally shown in the antisense strand of the human genome. FAM, fluorescein amidite; HEX, hexachlorofluorescein.

The clinical suspicion of HCL is typically triggered by pancytopenia (including monocytopenia), splenomegaly, and the presence in the blood smear of (usually few) hairy cells (ie, mature lymphoid cells with wide cytoplasm, no nucleoli, and thin circumferential projections; Fig 1, bottom right).^1^ Traditionally, the diagnosis is confirmed by documenting, through flow cytometry of a blood or marrow sample and/or immunohistochemistry of a bone marrow biopsy, coexpression of mature B-cell markers (eg, CD20 and CD22) together with CD11c, CD103, and CD25.^1^ More recently, genomic studies highlighting genes selectively expressed or mutated in HCL^10,20^ have been successfully translated in two new, excellent tools for confirming the diagnosis of HCL in general, and confirming the diagnosis in unusual anatomic sites in particular,^34^ as well as for distinguishing HCL from its mimickers. These tools are annexin-1 expression by immunohistochemistry, which is the most sensitive and specific immunophenotypic marker of HCL,^61,62^ and BRAF-V600E detection in HCL (but not HCL-like neoplasms) by molecular techniques on blood or marrow specimens (aspirates, smears, or fixed biopsies) or by immunohistochemical staining of fixed biopsies (Fig 2).^19,20,24-37^

In the diagnostic workup, HCL must be differentiated from other chronic mature B-cell tumors that share a similar clinicopathologic picture (splenomegaly without lymphadenopathy, some cytopenia, and circulating leukemic cells displaying some surface projections). These HCL-like neoplasms, which include HCL-variant, splenic marginal zone lymphoma, splenic diffuse red pulp small B-cell lymphoma, and other unclassifiable splenic lymphomas, represent specific entities (definitive or provisional) in the WHO classification of hematologic cancers,^41,53^ are characterized by other recurrent genetic lesions (Table 1), have a different and usually poorer prognosis compared with HCL, and do not respond well to purine analogs.

**Table 1.**
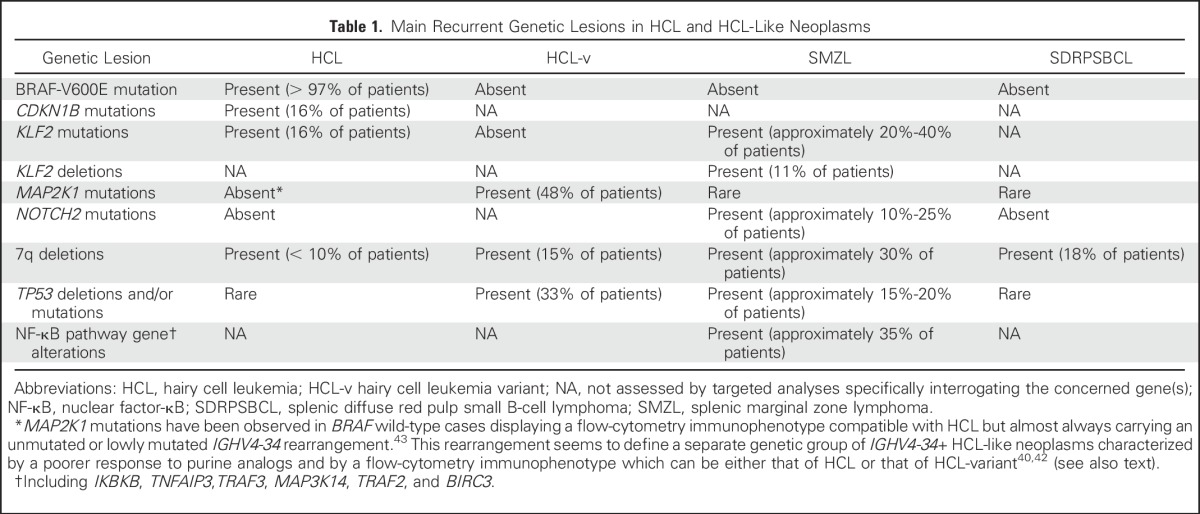
Main Recurrent Genetic Lesions in HCL and HCL-Like Neoplasms

Distinguishing HCL from its mimickers is of paramount clinical relevance and is greatly facilitated by detecting the BRAF-V600E mutation in HCL but not HCL-like tumors. This genetics-based differential diagnosis can even be obtained noninvasively in routine whole-blood samples. Because the latter often contain less than 10% HCL cells,^24^ it is crucial to use adequately sensitive techniques, such as allele-specific polymerase chain reaction (conventional or digital) and targeted deep sequencing (Fig 2).

## THE BRAF-V600E MUTATION AND HCL THERAPY

The current front-line standard of care in HCL is chemotherapy with cladribine or pentostatin.^63-65^ Both of these purine analogs induce complete responses (CRs) in approximately 85% of patients, usually lasting several years.^4^ With the aim of further improving on these results, the addition of the anti-CD20 monoclonal antibody rituximab to cladribine^66^ is currently being explored in a phase II randomized clinical trial in the United States (ClinicalTrials.gov identifier: NCT00923013).

However, up to 50% of patients experience relapse,^67,68^ and patients tend to respond progressively less well to rechallenge with purine analogs, unless rituximab is added to chemotherapy.^69^ Furthermore, the repeated use of chemotherapy can be aggravated by cumulative myelotoxicity and immunosuppression. In the relapsed or refractory setting, other less toxic, but also less effective, options include interferon alfa, rituximab monotherapy, and splenectomy.^4^ Among investigational therapies in this setting, the anti-CD22 immunotoxin moxetumomab pasudotox showed high clinical activity in a phase I trial (46% CR rate in 28 patients),^70^ and a confirmatory, pivotal, single-arm, phase III trial is ongoing internationally (ClinicalTrials.gov identifier: NCT01829711). Ibrutinib, an inhibitor of the Bruton tyrosine kinase that transduces the B-cell receptor signal, is also being explored in a multicenter single-arm phase II trial in the United States (ClinicalTrials.gov identifier: NCT01841723), with preliminary results at a dose of 420 mg daily showing a CR in one (9%) of 11 patients with relapsed HCL.^71^

The discovery of BRAF-V600E as the genetic cause of HCL,^20^ the development of oral BRAF or MEK inhibitors for *BRAF*-V600E–positive metastatic melanoma,^72^ and the preclinical studies strongly supporting the use of these inhibitors in HCL^21^ provide an important new approach to the therapy of patients with relapsed or refractory HCL.

First suggested in an anecdotal case,^73^ the clinical efficacy of the BRAF inhibitor vemurafenib was recently evaluated by two single-arm phase II multicenter trials, in Italy and the United States, in 25 and 24 patients with relapsed or refractory HCL, respectively.^22^ Vemurafenib, given at its standard dose of 960 mg twice daily for a median of 16 or 18 weeks, produced response rates of 96% and 100% in the two studies, of which 35% and 42% were CRs (Fig 1, middle right), respectively. In the Italian trial, with longer follow-up (median, 23 months), the median relapse-free survival time was 19 months after CR and 6 months after partial remission, and the median treatment-free survival times were 25 and 18 months, respectively.^22^ In both trials, toxicity was largely grade 1 or 2 in severity and, similarly to the melanoma setting,^74^ mostly consisted of rash, arthralgia, and cutaneous tumors of low invasive potential requiring a simple excision. Importantly, no significant myelotoxicity was observed, which also suggests the potential value of vemurafenib in other settings, for example in patients presenting with an opportunistic infection^75^ in whom myelotoxic chemotherapy is risky; vemurafenib might even be considered as front-line treatment in these patients.

The dramatic activity of vemurafenib in relapsed or refractory HCL,^22^ which might be obtained even with lower drug doses,^50^ proved to be far superior than in BRAF-V600E–positive melanoma (response rates of approximately 50%, almost always partial, and lasting a median of 7 months despite continuous drug intake until progression).^74^ BRAF-V600E–positive colorectal carcinoma is even altogether refractory to vemurafenib, probably as a result of epidermal growth factor receptor–mediated MEK-ERK reactivation bypassing mutant *BRAF* through RAF1/CRAF.^76^ This tremendous response variability stresses the paradigm of precision medicine by accentuating the level of precision required for drug effectiveness (an identical clonal genetic lesion translates into the right molecular target only if, and inasmuch as, the actual cellular context allows) and reinforces the preclinical need of thoroughly investigating the concerned signaling pathway(s) in each specific cell type of interest.^21^

**Fig 3.**
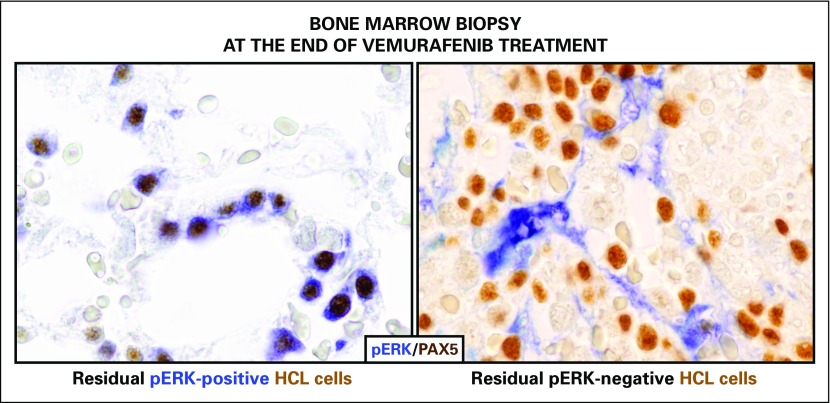
Resistance to vemurafenib in hairy cell leukemia (HCL). Shown are fixed bone marrow biopsies taken from patients with HCL the day after the end of vemurafenib treatment and double stained for the transcription factor PAX5 (a nuclear B-cell marker; in brown) and phospho-ERK (pERK; in blue). Residual leukemic cells (brown in both panels) persist in all patients, even in complete responders. However, in some cases (exemplified in the left panel), resistant HCL cells show ERK phosphorylation despite still being exposed to vemurafenib, suggesting the cells managed to rephosphorylate ERK through alternative bypass mechanisms. In other patients (exemplified in the right panel), resistant HCL cells do not detectably express pERK (with stromal cells being strongly pERK positive and thus acting as internal positive control), suggesting that they do not depend on strong ERK rephosphorylation for their continued survival. The micrographs in this figure are reprinted with permission.^22^ Copyright Massachusetts Medical Society.

However, relapse of HCL after a brief course of vemurafenib eventually ensues from residual bone marrow leukemic cells that persist at the end of treatment even in complete responders (Fig 1, middle) and that show, in approximately half of cases, bypass ERK rephosphorylation despite ongoing BRAF inhibition (Fig 3).^22^ The latter represents an acquired resistance mechanism that is also frequently operative in vemurafenib-treated patients with melanoma and is a result of a variety of cell-autonomous or microenvironmental cues (eg, mutant *BRAF* gene amplification or aberrant splicing; *MEK1* mutations; *RAS* mutations activating RAF1/CRAF; paracrine stimulation of receptor tyrosine kinases, also signaling through RAS-RAF1/CRAF).^77^ The specific causes of incomplete HCL cell eradication by vemurafenib have not been comprehensively dissected. Initial ex vivo studies of HCL cells suggest that bone marrow stromal cells can counteract vemurafenib-induced MEK-ERK dephosphorylation and apoptosis, pointing to microenvironment-mediated adaptive resistance.^21^ However, in one patient with HCL who experienced relapse after vemurafenib, resistance to vemurafenib rechallenge was linked to two newly acquired subclonal activating *KRAS* mutations.^22^ In any case, it should be kept in mind that, in contrast to continuous vemurafenib dosing until progression as done in melanoma, patients with HCL received a shorter course of the drug with a fixed duration.^22^ Thus, the escape mechanisms in the HCL clone eventually re-emerging at relapse (after several months, or even a few years, of relief from the selective pressure of BRAF inhibition) might be different from, and possibly less pronounced than in, melanoma. Indeed, at HCL relapse after vemurafenib, retreatment with the same drug was usually able to elicit second responses, although they tended to be less profound and less durable.^22^

Two strategies are currently being pursued to counteract vemurafenib resistance in HCL. An international phase II basket trial on BRAF-V600E–positive rare cancers, including HCL (ClinicalTrials.gov identifier: NCT02034110), is testing combined BRAF and MEK blockade, which opposes the ERK-rephosphorylating bypass mechanisms (Fig 3, left) and has already proved to be more effective than BRAF inhibition alone in BRAF-V600E–positive patients with melanoma.^78^ In the latter patients, this approach also considerably reduced the incidence of skin tumors, which are a result of paradoxic RAF1/CRAF-MEK-ERK signaling triggered by BRAF inhibitors in *BRAF* wild-type keratinocytes or melanocytes with pre-existing RAS activation.^79^ The second strategy is to attack vemurafenib-resistant cells, irrespective of their dependence or independence on MEK-ERK resphosphorylation (Fig 3), by adding to vemurafenib another targeted nonmyelotoxic agent, rituximab, which has a completely different, mainly immunologic, mechanism of action. This strategy is being tested in a phase II Italian trial (EudraCT 2014-003046-27).
